# Morphology Effect of Puffball Spores on Hemostasis: A Promising Solution for Hemostatic Challenges

**DOI:** 10.1002/advs.202417566

**Published:** 2025-02-28

**Authors:** Xuechang Pei, Yue Feng, Yanru Wu, Jie Zhang, Jianlan Li, Shutai Jiang, Huijun Huang, Ping Qin, Guoqing Li, Xinrui Guo, Mingxian Liu, Chuanxi Wang, Hao Gao

**Affiliations:** ^1^ Institute of Traditional Chinese Medicine & Natural Products College of Pharmacy State Key Laboratory of Bioactive Molecules and Druggability Assessment International Cooperative Laboratory of Traditional Chinese Medicine Modernization and Innovative Drug Development of Ministry of Education (MOE) of China Guangdong Province Key Laboratory of Pharmacodynamic Constituents of TCM and New Drugs Research Jinan University Guangzhou Guangdong 510632 P. R. China; ^2^ Department of Materials Science and Engineering College of Chemistry and Materials Science Jinan University Guangzhou Guangdong 511443 P. R. China

**Keywords:** hemostatic materials, hollow ball‐rod morphology, nano protrusions, pinning effect, puffball spores

## Abstract

Hemostatic materials play a crucial role in wound healing by promoting blood concentration or releasing procoagulant factors. While hydrophilic hemostatic materials are effective, they may cause excessive blood loss and difficulty removing from the wound. Conversely, hydrophobic hemostatic materials avoid these issues but may hinder blood concentration and the release of procoagulant factors due to their water‐repellent nature. This study investigates the hemostatic properties and underlying mechanism of puffball (*Bovistella* sp.) spores, a traditional hemostatic material. The unique hollow ball‐rod morphology and strong water affinity of puffball spores enable efficient water removal, leading to improved blood clotting without the drawbacks typically associated with hydrophilic hemostatic materials. Further analysis reveals that the nano‐protrusions on the spore surface create a textured hydrophobic surface due to the pinning effect, which prevents adhesion to the wound after clotting. Overall, puffball spores exhibit hemostatic efficacy comparable to the commercial agent QuikClot, with enhanced safety and reduced side effects. Their characteristic morphology, physicochemical properties, and chemical compositions offer inspiration for advancing hemostatic materials and addressing current challenges in wound healing. Additionally, this work provides new perspectives for insight into the pharmacological substance basis of traditional medicine, expanding beyond the conventional component‐focused mentality to a material‐based insight.

## Graphical abstract

This work presents an innovative approach to address current challenges in hemostasis by drawing inspiration from the natural hemostatic material, Puffball spores (*Bovistella* sp.). Their unique hollow ball‐rod morphology and texture composition enable blood concentration and promote the release of procoagulant factors, while the nano‐protrusions on the surface effectively prevent unwanted wound adhesion after coagulation.

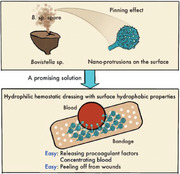

## 1 Introduction

Uncontrollable hemorrhage is a leading cause of death following trauma, particularly in cases involving traffic accidents, natural disasters, and wars.^[^ ^1^ ^]^ Hemostatic dressings are commonly used to reduce excessive blood loss by absorbing water from blood,^[^ ^2^ ^]^ releasing procoagulant factors,^[^ ^3^ ^]^ and sealing the wound.^[^ ^4^ ^]^ Hydrophilic hemostatic dressings are effective in blood concentration and releasing procoagulant factors. Among them, QuikClot Combat Gauze is recommended by the Committee on Tactical Combat Casualty Care as the preferred hemostatic agent for U.S. military use.^[^ ^5^ ^]^ However, they have side effects such as difficulty in removal from wounds and risk of infections.^[^ ^6^ ^]^ On the other hand, hydrophobic hemostatic dressings can effectively overcome these side effects^[^ ^7^ ^]^ and prevent excessive blood loss.^[^ ^8^ ^]^ Such unique advantages have garnered much attention from researchers.^[^ ^9^ ^]^ Yet, despite great efforts, hydrophobic hemostatic dressings and other easy‐to‐peel dressings remain scarcely reported. These dressings are primarily constructed by incorporating a hydrophobic layer to create Janus structures or by loading hydrophobic materials onto the substrate's surface.^[^ ^10^ ^]^ For instance, Smith & Nephew Opsite dressing and Mannings adhesive waterproof dressing serve as examples of Janus‐structured systems, with their wound contact layer made of a porous hydrophobic film.^[^ ^[Link]^, ^[Link]^ ^]^ The 3 M Nexcare DUO is a Janus‐structured system, featuring a hydrophobic film as its wound contact layer.^[^ ^6a^ ^]^ Recently, several studies have reported on the incorporation of hydrophobic materials onto substrate surfaces for the development of hydrophobic hemostatic dressings. For instance, in 2018, a hemostatic fabric material with hydrophobic properties was constructed by coating paraffin on cotton gauze.^[^ ^11^ ^]^ In 2019, Yap et al. developed a hydrophobic hemostatic dressing by immobilizing carbon nanofibers on cotton gauze.^[^ ^6a^ ^]^ In 2024, a hydrophobic hemostatic gauze with polydimethylsiloxane and hydrophobic‐modified cellulose nanocrystals loaded on the surface was developed.^[^ ^6b^ ^]^ While hydrophobic hemostatic dressings address some concerns, they struggle with blood concentration and releasing procoagulant factors due to their water‐repellent nature.^[^ ^12^ ^]^ An effective solution to address the limitations of hydrophobic hemostatic dressings has yet to be identified. Developing hemostatic dressings with better wound‐healing properties and minimal side effects remains challenging.^[^ ^13^ ^]^

**Figure 1:**
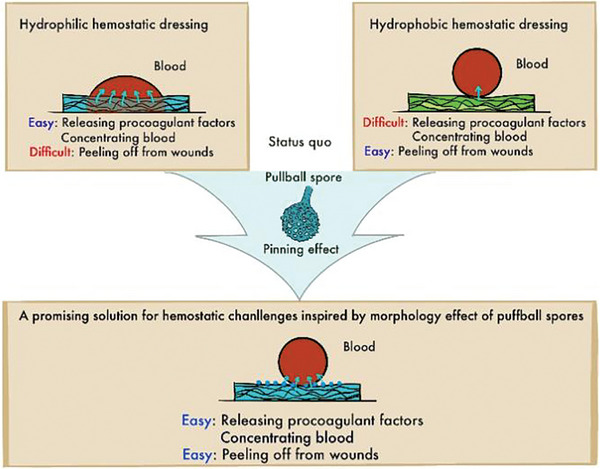
Schematic diagram of the current status of hemostatic dressings and the innovation of this study.

Nature is a treasure trove of hemostatic materials. For example, halloysite, carbonized human hair,^[^ ^14^ ^]^ *Bletilla striata* charcoal,^[^ ^15^ ^]^ and puffball have all demonstrated hemostatic effects. Puffball spore, in particular, a well‐known traditional natural medicine, has been extensively utilized for wound healing for centuries due to its exceptional hemostatic effects.^[^ ^16^ ^]^ Although the exoskeleton of puffball spores is composed of chitin, a hydrophilic polysaccharide polymer, puffball spores, especially those from *Bovistella* species (*B*. sp.), exhibit apparent hydrophobic behavior and can be easily exfoliated from wounds. This led us to investigate how the surface morphology of puffball spores influences their hemostatic effectiveness. The hollow ball‐rod structure of puffball spores is conducive to water adsorption, facilitating blood concentration. While the nanoscale protrusions on the external surface render the texture hydrophobic, preventing adhesion after coagulation. Further experiments were conducted to confirm the high water affinity and adsorption capacity of puffball spores, and the hydrophobic behavior was attributed to the pinning effect of nano‐protrusions on the surface (**Figure** **1**). In addition, the hemostatic mechanism and biocompatibility of puffball spores are also discussed.

## 2 Results and Discussion

### 2.1 The Hemostatic Efficacy of *B*. sp. Spores and its Dressing

**Figure 2:**
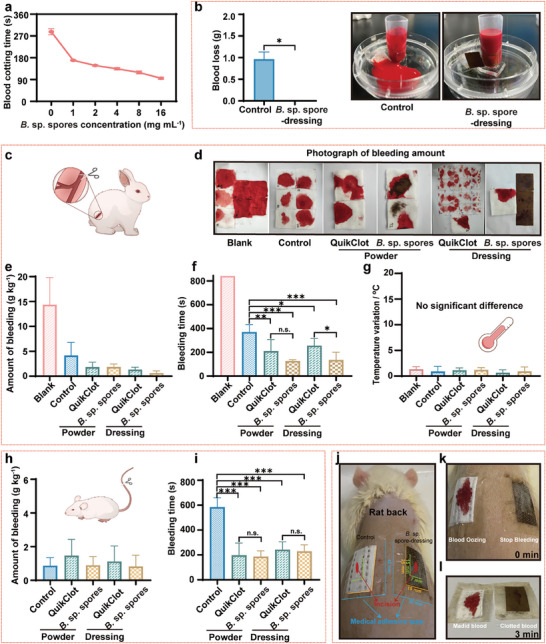
In vivo, the hemostatic capability of *B*. sp. spores and *B*. sp. spore‐dressing. a) Blood clotting time of *B*. sp. spores at different concentrations. Data are presented as mean ± SD (*n* = 3). b) Blood loss of control and *B*. sp. spore‐dressing in a vascular injury model: photographs and blood loss bar graph of control and *B*. sp. spore‐dressing at 13 min, respectively. Data are presented as mean ± SD (*n* = 3). *p < 0.05 by unpaired student's *t*‐test. c–g) In vivo hemostatic capability of *B*. sp. spores and *B*. sp. spore‐dressing in rabbit femoral arteriovenous injury model: (c) schematic diagram, (d) representative photographs, (e) histograms of total blood loss amounts, (f) histograms of hemostasis time, and (g) temperature variation. Data are presented as mean ± SD (*n* = 6). Data are compared separately by one‐way ANOVA (analysis of variance) using a Tukey post‐hoc analysis. n.s. = not significant, *p < 0.05, **p < 0.01, ***p < 0.001 denote statistically significant variation. h, i) In vivo hemostatic capability of *B*. sp. spores and *B*. sp. spore‐dressing in rat tail amputation model: (h) histograms of total blood loss amounts and (i) histograms of hemostasis time. Data are presented as mean ± SD (*n* = 5). Data are compared separately by one‐way ANOVA (analysis of variance) using a Tukey post‐hoc analysis: n.s. = not significant, ***p < 0.001 denotes statistically significant variation. j‐l) In vivo hemostatic capability of *B*. sp. spore‐dressing in rat tangential wound model: (j) schematic diagram of two different dressings on the rat back. (k) Photographs of control and *B*. sp. spore‐dressing applied on the wounds at 0 min. (l) Photographs of control and *B*. sp. spore‐dressing applied on the wounds at 3 min. (*n* = 3).

Although puffball (*B*. sp.) spores have long been used for hemorrhage control, their hemostatic efficacy was rigorously demonstrated for the first time in this study through in vitro and in vivo hemostasis models. One challenge with using puffball spores is that they are in powder form and can be easily dispersed by blood flow. To address this issue, we developed a hemostatic patch dressing and systematically evaluated its effectiveness in hemostasis. First, blood clotting assays were conducted to reveal that *B*. sp. spores can indeed expedite blood coagulation. At a low concentration of 1 mg mL^−1^, the blood clotting time was reduced by over 40% compared to the natural process (287 ± 13 s), and it can be further reduced with increasing concentration of *B*. sp. spores (**Figure** **2**a). Second, the *B*. sp. spore‐dressing (20% w/w ratio of *B*. sp. spores to Basewing dressing, as shown in Figure S1a, Supporting Information) was fabricated and examined in a vascular injury model, showing improved blood coagulation and diminished blood outflow. As depicted in Figure 2b, the mean blood loss in the *B*. sp. spore‐dressing group was significantly reduced (0.0 g) compared to the control group (0.96 ± 0.17 g). Moreover, no blood adhesion was observed on the contact surface. Thirdly, the hemostatic performance of *B*. sp. spores and *B*. sp. spore‐dressing was assessed using three distinct animal models: the rabbit femoral arteriovenous injury model, the rat tail amputation model, and the rat tangential wound model. In the rabbit model of femoral arteriovenous injury (Figure 2c–g; Videos S1–S6, Supplementary Video), the duration of bleeding for the *B*. sp. spores treated group (125 ± 12 s) was significantly reduced compared to the blank group (over 840 s) and the commercial product QuikClot powder (208 ± 100 s). Similarly, the duration of bleeding for the group treated with *B*. sp. spore‐dressing (133 ± 67 s) was markedly less than that of the control group (368 ± 65 s) and the QuikClot Combat Gauze (254 ± 62 s). Furthermore, the total amounts of bleeding treated with *B*. sp. spores (1.86 ± 0.59 g) and *B*. sp. spore‐dressing (0.59 ± 0.47 g) were significantly lower than that of the control group (4.16 ± 2.62 g). There was no statistically significant difference when compared to QuikClot powder (1.82 ± 1.01 g) and QuikClot Combat Gauze (1.32 ± 0.50 g), respectively. Similarly, in the rat tail amputation model, *B*. sp. spores and *B*. sp. spore‐dressing showed improved hemostasis compared to control group and commercial products (Figure 2h,i). The bleeding time of the group treated with *B*. sp. spores (185 ± 48 s) was significantly reduced compared to the control group (583 ± 76 s) and in the same range compared to QuikClot powder (196 ± 99 s). Furthermore, the bleeding time treated with *B*. sp. spore‐dressing (227 ± 54 s) was comparable to that of QuikClot Combat Gauze (240 ± 66 s). Moreover, in the rat tangential wound model, the application of *B*. sp. spore‐dressing for epidermal trauma (Figure 2j–l; Figure S2, Supporting Information) showed a substantial reduction in blood loss compared to the control group. These results underscore the effectiveness of *B*. sp. spores and *B*. sp. spore‐dressing in efficiently arresting hemorrhage in various animal models, suggesting that *B*. sp. spores could be a promising approach for managing bleeding in clinical settings.

### 2.2 The Characterization of *B*. sp. Spores

**Figure 3:**
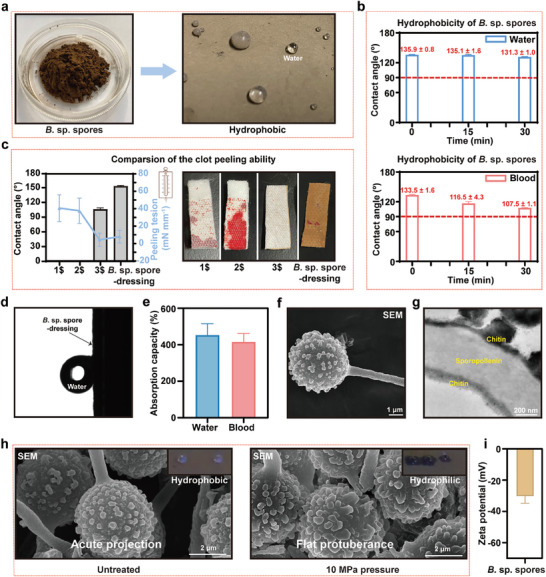
Characterization of *B*. sp. spores a) Photograph of *B*. sp. spores and water droplets on their surfaces. b) Water and blood contact angles of *B*. sp. spores. Data are presented as mean ± SD (*n* = 3). c) Comparison and photograph of the clot peeling tension of *B*. sp. spore‐dressing with “easy‐to‐peel” commercial dressings: Smith & Nephew OPSITE dressing (1$), Mannings adhesive waterproof dressing (2$), and 3 M Nexcare DUO dressing (3$). Data are presented as mean ± SD (*n* = 3). d) Evaluation of the dynamic contact angle of *B*. sp. spores in water (rotated to 90°). e) Water and blood absorption capacities of *B*. sp. spores in histograms. Data are presented as mean ± SD (*n* = 3). f) FE‐SEM image of *B*. sp. spore. g) TEM image of *B*. sp. spore wall structure. h) Comparison of *B*. sp. spores before and after compression at 10 MPa. i) Zeta potential of *B*. sp. spores. Data are presented as mean ± SD (*n* = 6).

As discussed above, both *B*. sp. spores and *B*. sp. spore‐dressing demonstrated significant hemostatic efficacy and exhibited non‐adhesion behavior toward wounds. To understand the underlying reasons behind this, a detailed analysis of the structural morphology, physicochemical properties, and chemical composition of *B*. sp. spores was conducted. Water droplets on *B*. sp. spores displayed a spherical rolling behavior (**Figure** **3**a), and both water and blood droplets exhibited static contact angles exceeding 90° for at least 30 min (Figure 3b), indicating surface hydrophobicity of *B*. sp. spores. This hydrophobicity behavior of the dressing's surface is beneficial for its stripping from the wound sites. To assess the ease of peeling off the *B*. sp. spore‐dressing from the wound post‐coagulation, a clot peeling tension test was performed and compared with three commercial easy‐to‐peel dressings: Smith & Nephew Opsite dressing (1$), Mannings adhesive waterproof dressing (2$), and 3 M Nexcare DUO dressing (3$), using 3 M artificial skin (3M Tegaderm hydrocolloid thin dressing) as a substrate. The results revealed that the *B*. sp. spore‐dressing outperformed the 1$ and 2$ dressings and exhibited a peeling ability comparable to the 3$ dressing (Figure 3c). Surprisingly, during the rolling test, the water droplets remained on the contact surface even when rotated to 90°, indicating significant adhesion (Figure 3d). Additionally, despite appearing water resistant, *B*. sp. spores eventually absorbed water or blood ≈4 times their weight after extended exposure (Figure 3e; Videos S7–S10, Supplementary Video). Further electron microscopy and atomic force microscopy analyses of *B*. sp. spores revealed a hollow ball‐rod‐like morphology with surface protrusions (Figure 3f; Figure S3a,b, and Table S1, Supporting Information). The dimensions of the spherical parts and protrusive components were ≈4 µm and 250 nm, respectively. Ultrathin sectioning, in combination with transmission electron microscopy (TEM) and confocal microscopy, was utilized to elucidate the structural composition of the spore wall with fluorescent brightener 28 and auramine O staining techniques. As depicted in Figure 3g and Figure S3c–f (Supporting Information), the spore wall was found to be composed of three distinct layers with the exterior and interior layers (1# and 3#) predominantly consisting of chitin, and the middle section (2#) is mainly sporopollenin. The presence of chitin on the exterior layer contributes to the strong water adhesion due to hydrogen bonding with water, a commonly observed feature in fungal spores that facilitate water absorption during their transition from dormancy to germination.^[^ ^17^ ^]^ Additionally, the hollow spherical framework of *B*. sp. spores, along with the hydrophilic chitins in the exterior and interior layers, results in enhanced water adsorption capacity. Interestingly, although the outer wall of *B*. sp. spores is composed of hydrophilic chitin, it exhibits hydrophobic behavior when in contact with water or blood. We speculated that this peculiar phenomenon may be attributable to the pinning effect generated by the nano‐protrusions on the surface. To verify our speculation, water contact angle tests were conducted on the *B*. sp. spores with the nano‐protrusions destroyed by 10 MPa pressure, revealing a clear shift from hydrophobicity to hydrophilicity, as evidenced by the quick soaking of water instead of forming water droplets (Figure 3h). Furthermore, chemical composition analysis with infrared spectroscopy revealed the presence of polysaccharides, lipids, and proteins (Figure S3g), and optical emission spectroscopy analysis detected the presence of calcium, magnesium, and iron in puffball spores (Table S2, Supporting Information). The zeta (ζ) potential of the *B*. sp. spores was measured to be ≈−30.2 ± 4.6 mV (Figure 3i). It is worth noting that calcium, magnesium, and iron ions can act as procoagulant factors in the blood,^[^ ^18^ ^]^ and negative charges can activate the intrinsic coagulation pathway,^[^ ^19^ ^]^ both of which play crucial roles in promoting hemostasis.

### 2.3 The Hemostatic Mechanism of *B*. sp. Spores

**Figure 4:**
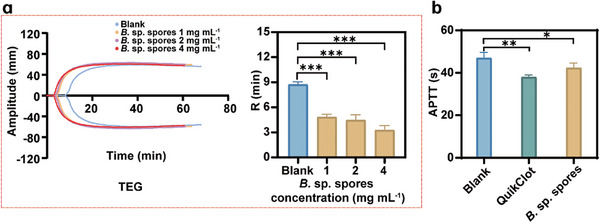
Hemostatic mechanism of *B*. sp. spores. a) TEG results of *B*. sp. spores and histograms of R‐value. Data are presented as mean ± SD (*n* = 3). Data are compared separately by one‐way ANOVA (analysis of variance) using a Tukey post‐hoc analysis. ***p < 0.001 denotes statistically significant variation. b) APTT results of *B*. sp. spores. Data are presented as mean ± SD (*n* = 3). Data are compared separately by one‐way ANOVA (analysis of variance) using a Tukey post‐hoc analysis. *p < 0.05, **p < 0.01 denote statistically significant variation.

To investigate the hemostatic mechanism of *B*. sp. spores, thromboelastometry (TEG) and activated partial thromboplastin time (APTT) assays were conducted. The TEG experiments revealed that the application of *B*. sp. spores led to a substantial decrease in the R‐value, a coagulation reaction time reflecting coagulation activity. As illustrated in **Figure** **4**a and detailed in Table S3 (Supporting Information), the group treated with *B*. sp. spores group at a concentration of 4 mg mL^−1^ (R‐value: 3.27 ± 0.55 min) showed significantly enhanced coagulation performance compared to the blank group (R‐value: 8.73 ± 0.32 min). This suggests that *B*. sp. spores may facilitate the activation of coagulation factors, leading to faster hemostasis. In the APTT assay, as illustrated in Figure 4b, the administration of *B*. sp. spores resulted in a reduced APTT value (42.3 ± 2.3 s) compared to the blank group (47.0 ± 2.7 s), indicating activation of the intrinsic coagulation pathway by *B*. sp. spores.

### 2.4 The Biocompatibility of *B*. sp. Spores and its Dressing

**Figure 5:**
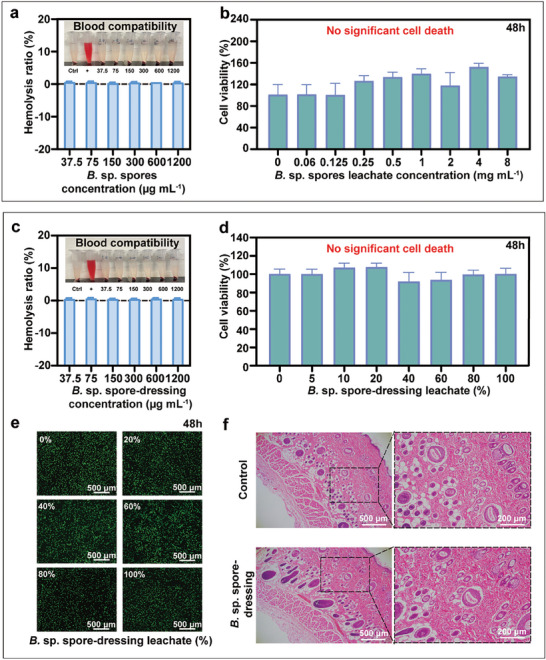
Biocompatibility of *B*. sp. spores and *B*. sp. spore‐dressing. a) Blood compatibility of *B*. sp. spores. Data are presented as mean ± SD (*n* = 3). b) Cell viability assay of *B*. sp. spores on L929 cells. Data are presented as mean ± SD (*n* = 6). Data are compared separately by one‐way ANOVA (analysis of variance) using Tukey post‐hoc analysis. c) Blood compatibility of *B*. sp. spore‐dressing. Data are presented as mean ± SD (*n* = 3). d) Cell viability assay of *B*. sp. spore‐dressing on L929 cells. Data are presented as mean ± SD (*n* = 6). Data are compared separately by one‐way ANOVA (analysis of variance) using Tukey post‐hoc analysis. e) AO/EB live/dead cell staining of L929 cell compatibility test. f) H&E staining of skin sections after 24 h of control and *B*. sp. spore‐dressing exposure.

The biocompatibility of *B*. sp. spores and *B*. sp. spore‐dressing is critical due to their direct contact with wounds as hemostatic agents. Thus, blood compatibility tests, cell viability assays, and skin irritation experiments were performed using both in vitro and in vivo models (**Figure** **5**). The results consistently demonstrated that both *B*. sp. spores and *B*. sp. spore‐dressing are highly biocompatible. Furthermore, some hemostatic materials have been associated with temperature alterations that may result in thermal injury to wounds.^[^ ^20^ ^]^ Consequently, we evaluated the temperature variations in a rabbit femoral arteriovenous injury model. The results showed that the application of *B*. sp. spores did not lead to any significant fluctuations in wound temperature (Figure 2g).

In summary, *B*. sp. spore‐dressing has demonstrated a hemostatic efficacy comparable to the commercially available QuikClot Combat Gauze, while maintaining a satisfying safety profile. They do not adhere to wounds, causing excessive blood loss, or generating local temperature increases that could potentially burn the wound. These positive attributes can be attributed to their unique structural characteristics, physicochemical properties,^[^ ^21^ ^]^ and chemical composition of *B*. sp. spores. Specifically, i) the outer wall of *B*. sp. spores is composed of hydrophilic chitin, which has a high affinity for water, facilitating the interaction with blood and promoting the release of procoagulant factors. *B*. sp. spores contain certain metal ions, such as Ca^2+^, Mg^2+^, and Fe^3+^, which play crucial roles in the coagulation process and are recognized as key procoagulant factors. ii) The hollow, micro‐nano‐scale ball‐rod‐like structure of *B*. sp. spores allows for absorbing large amounts of water and subsequent blood concentration. This distinctive structural feature also increases the contact surface area with blood, enhancing the hemostatic effect. iii) The negative charges on the surface of *B*. sp. spores assist the process of hemostasis, promoting faster blood clotting by initiating the intrinsic coagulation pathway. iv) The nano‐protrusions on the surface of *B*. sp. spores create a pinning effect, imparting the surface with hydrophobic properties that can prevent adhesion to the wounds and reduce blood loss by forming physical barriers.

## 3 Conclusion

The advancement of hydrophobic hemostatic materials has become a focal point for future research due to their potential to avoid the side effects associated with hydrophilic counterparts. Yet, their water‐repellent nature may hinder blood concentration and procoagulant factor release. This study provides novel perspectives and valuable insights for addressing the challenges in developing hemostatic materials for medical applications. Through a detailed investigation of *B*. sp. spores, a traditional natural hemostatic material, we have discovered that the hydrophilic outer layer of the hollow structure shows strong water affinity, facilitating water removal and enhancing blood concentration, while the unique surface nano‐protrusions create a pinning effect, resulting in a hydrophobic surface texture that can prevent adhesion to the wounds. The pinning effect typically refers to the influence exerted by microstructures, whether they are intrinsic to the material or present on its surface, on the macroscopic physicochemical properties of the material. This phenomenon is a fundamental and widely employed strategy in the functional design and engineering of materials.^[^ ^22^ ^]^ Inspired by the inherent hemostatic efficacy of *B*. sp. spores, we propose the potential of tuning the surface morphology of hydrophilic hemostatic materials to enhance their hemostatic properties while minimizing adverse effects, such as wound adhesion and excessive blood loss.

This study specifically underscores the critical role of molecular assembly in influencing efficacy, highlighting the dynamic interaction between structural morphology and biological activity. In general, previous research has usually focused on the chemical composition of naturally occurring substances in the search for pharmacological substance basis of traditional medicine, including small molecules, peptides, proteins, polysaccharides, miRNA, and so on.^[^ ^23^ ^]^ This study posits that, in addition to the active chemical constituents, other factors such as physical architecture and surface morphology could significantly affect the overall effectiveness. Consequently, this concept allows us to think outside known limits and restrictions in studying traditional medicines, expanding beyond the conventional component‐focused mentality to a material‐based understanding of their biological efficacy. The findings in this study contest the perspective that efficacy is comprehended only at the molecular level, advocating a holistic and multi‐dimensional approach to assessing efficacy.

In addition, deciphering the principles behind natural phenomena is crucial for advancing biomimetic research. Bird wing mechanics inform aircraft design and bat echolocation inspires radar systems. Unraveling the mechanism by which puffball spores achieve hemostasis without adhering to wounds could provide a promising solution for developing non‐adhesive hemostatic materials. Furthermore, we envision the possibility of engineering advanced bionic hemostatic materials using progressively sophisticated micro‐nano 3D printing and etching techniques.^[^ ^24^ ^]^ In recent years, Tao et al. reported the development of a voltage‐regulated 3D electron beam lithography (EBL) technique capable of fabricating arbitrary 3D nanostructures with a resolution of less than 15 nm, enabling high‐resolution nanoscale 3D printing.^[^ ^25^ ^]^ Meanwhile, Wang et al. devised an innovative 3D printing strategy tailored for polysaccharide‐based materials, addressing challenges in fabricating polysaccharide scaffolds with improved shape fidelity.^[^ ^26^ ^]^ The aforementioned research advancements and technological innovations render it feasible to develop bionic hemostatic materials utilizing micro‐nano 3D printing and etching technology, employing puffball (*B*. sp.) spores as a template. In future studies, we will continue to investigate the structure‐activity relationship of hemostatic materials by systematically engineering micro/nanostructured substrates with tailored surface morphologies.

## 4 Experimental Section

All materials and methods can be found in Supporting Information.

## Conflict of Interest

The authors declare no conflict of interest.

## Supporting information



Supporting Information

Supplementary Video1

Supplementary Video2

Supplementary Video3

Supplementary Video4

Supplementary Video5

Supplementary Video6

Supplementary Video7

Supplementary Video8

Supplementary Video9

Supplementary Video10

## Data Availability

The data that support the findings of this study are available from the corresponding author upon reasonable request.
