# Supplementary material for: Morphology Effect of Puffball Spores on Hemostasis: A Promising Solution for Hemostatic Challenges
Source: Adv Sci (Weinh). 2025 Feb 28;12(16):2417566. doi: 10.1002/advs.202417566 (PMC12021107; doi:10.1002/advs.202417566)
Supplement: Supplementary file 1 — Supporting Information [file ADVS-12-2417566-s007.docx]

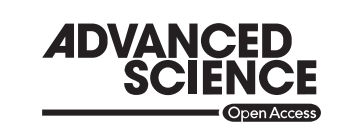


Supporting Information

for *Adv.Sci.*, DOI: 10.1002/advs.202417566

Morphology Effect of Puffball Spores on Hemostasis: A Promising Solution for Hemostatic Challenges

*Xuechang Pei, Yue Feng, Yanru Wu, Jie Zhang, Jianlan Li, Shutai Jiang, Huijun Huang, Ping Qin, Guoqing Li, Xinrui Guo, Mingxian Liu*, Chuanxi Wang* and Hao Gao**

**Supporting Information**

**Morphology Effect of Puffball Spores on Hemostasis: A Promising Solution for Hemostatic Challenges**

*Xuechang Pei, Yue Feng, Yanru Wu, Jie Zhang, Jianlan Li, Shutai Jiang, Huijun Huang, Ping Qin, Guoqing Li, Xinrui Guo,* *Mingxian Liu*,* *Chuanxi Wang*, Hao Gao**

*Correspondence to: Hao Gao and Chuanxi Wang

Institute of Traditional Chinese Medicine & Natural Products, College of Pharmacy / State Key Laboratory of Bioactive Molecules and Druggability Assessment / International Cooperative Laboratory of Traditional Chinese Medicine Modernization and Innovative Drug Development of Ministry of Education (MOE) of China / Guangdong Province Key Laboratory of Pharmacodynamic Constituents of TCM and New Drugs Research, Jinan University, Guangzhou, Guangdong 510632, P.R. China

E-mail: tghao@jnu.edu.cn; tcxwang@jnu.edu.cn

*Correspondence to: Mingxian Liu

Department of Materials Science and Engineering, College of Chemistry and Materials Science, Jinan University, Guangzhou, Guangdong 511443, P.R. China

E-mail: liumx@jnu.edu.cn

**This PDF file includes:**

Figures S1 to S5

Tables S1 to S3

Supplementary results

Supplementary materials and methods

Statistical analysis

References

**Supplemental figures**


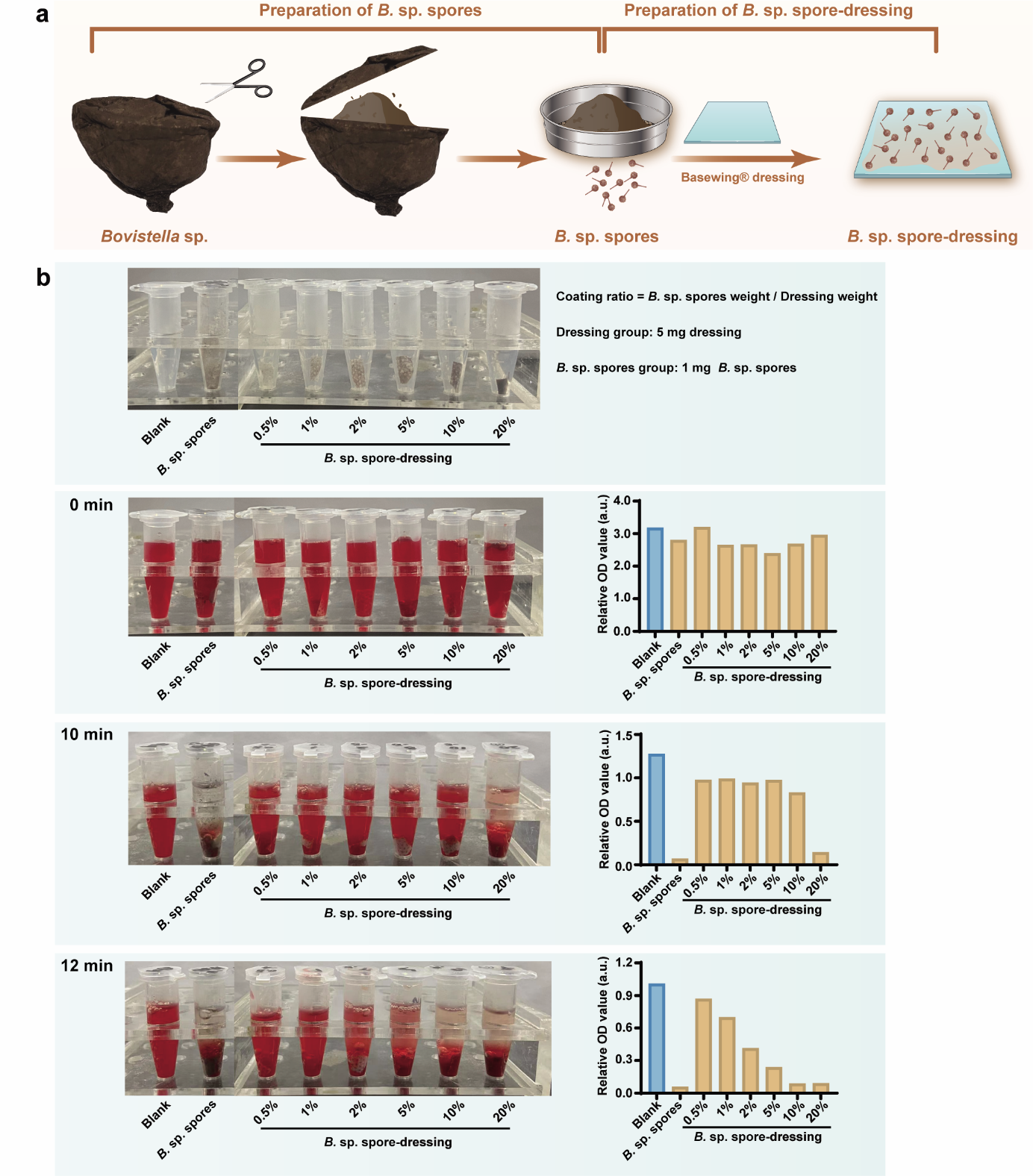


**Figure S1. Preparation and determination of the coating ratio of *B.* sp. spore-dressing.**

(a) Preparation of *B.* sp. spores and *B.* sp. spore-dressing. (b) Determine the coating ratio of *B.* sp. spore-dressing: photographs from the in vitro blood-clotting measurement and the corresponding relative OD values of the supernatant absorbance for blank, *B.* sp. spores (1mg) or *B.* sp. spore-dressings (5 mg) varied in weight rotio of *B.* sp. spores / dressing, ranging from 0.5%, 1%, 2%, 5%, 10%, and 20%, respectively.


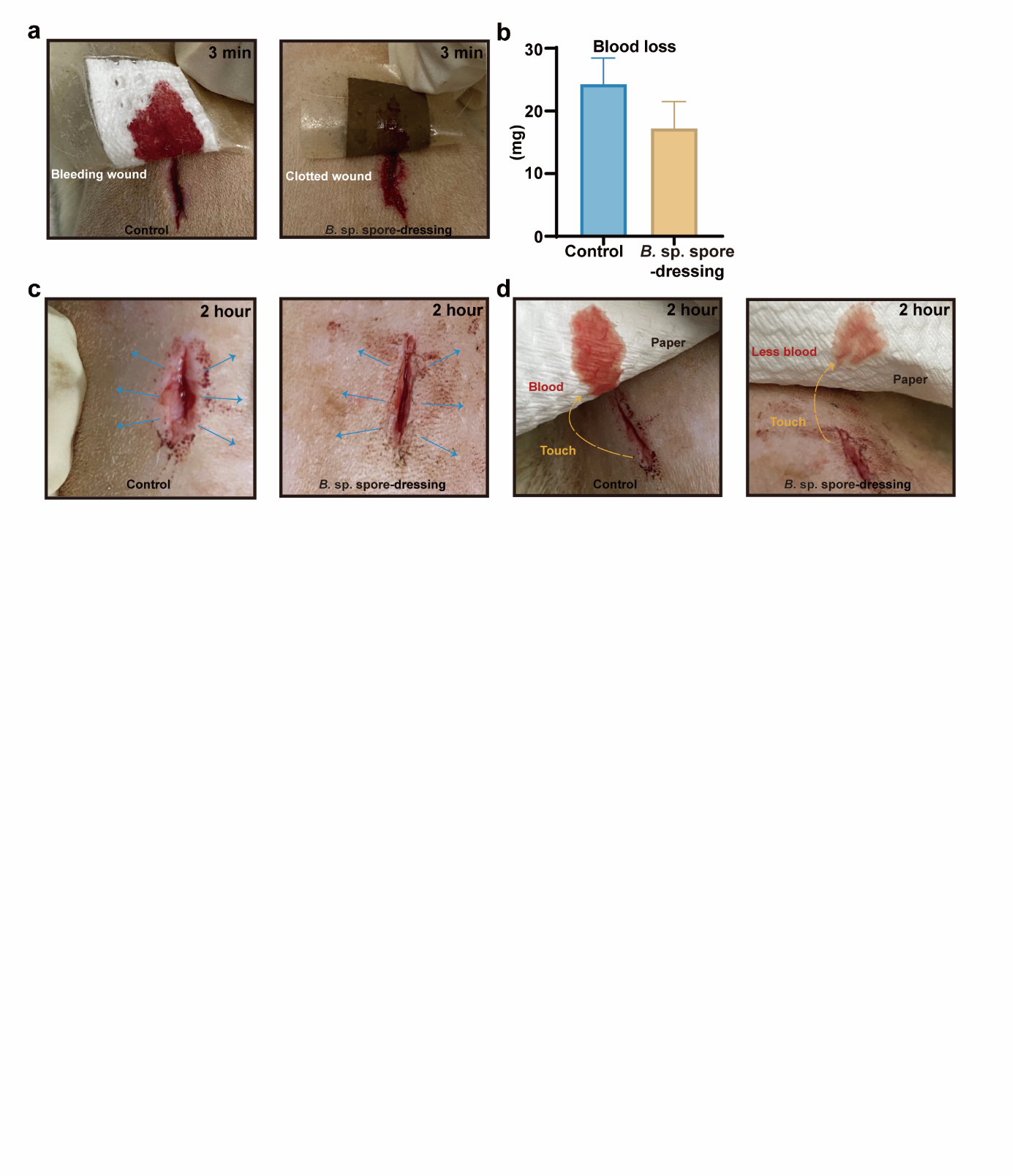


**Figure S2. In vivo hemostatic capability of *B.* sp. spore-dressing in rat tangential wound model.**

(a) Comparison of bleeding between dressing and *B.* sp. spore-dressing at 3 min; (b) Comparison of bleeding amount between dressing and *B.* sp. spore-dressing at 3 min. Data are presented as mean ± SD (*n* = 3). Data are compared by unpaired student’s *t*-test. (c) Photographs of wounds at 2 h; (d) Photographs of the secondary bleeding situation at 2 h.


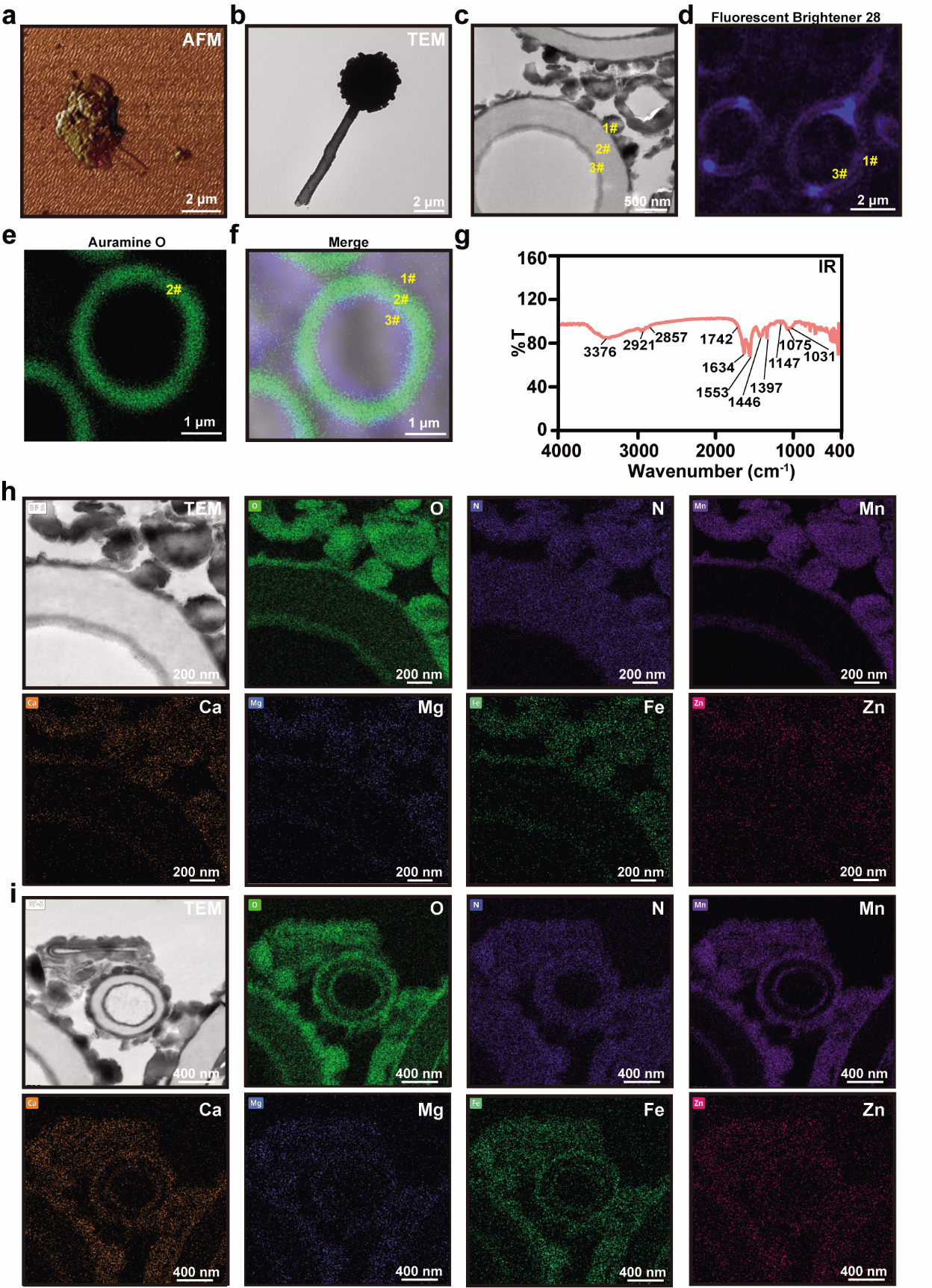


**Figure S3. Characterization of *B.* sp. spores structure.**

(a) Atomic force microscopy (AFM) image of *B.* sp. spore. (b) Transmission electron microscopy (TEM) image of *B.* sp. spore. (c) TEM image of *B.* sp. sporewall structure. (d) Confocal image of *B.* sp. spores stained with fluorescent whitening agent 28 for 16h. The blue ring presents the position of chitin on *B.* sp. spores (405 nm excitation / 422 nm emission). (e) Confocal image of *B.* sp. spores subjected to sequential staining with fluorescent whitening agent 28 for 16 h and auramine O for 10 min. The green ring presents the position of sporopollenin on *B.* sp. spores (498 nm excitation / 517 nm emission). (f) Merge confocal images of *B.* sp. spores subjected to sequential staining with fluorescent whitening agent 28 for 16 h and auramine O for 10 min. The blue ring presents the position of chitin on *B.* sp. spores (405 nm excitation / 422 nm emission, layers 1# and 3#). The green ring presents the position of sporopollenin on *B.* sp. spores (498 nm excitation / 517 nm emission, layer 2#). (g) IR spectrum of *B.* sp. spores. (h) EDS mapping analysis image of the ball-like part of *B.* sp. spore. (i) EDS mapping analysis image of the rod-like part of *B.* sp. spore.


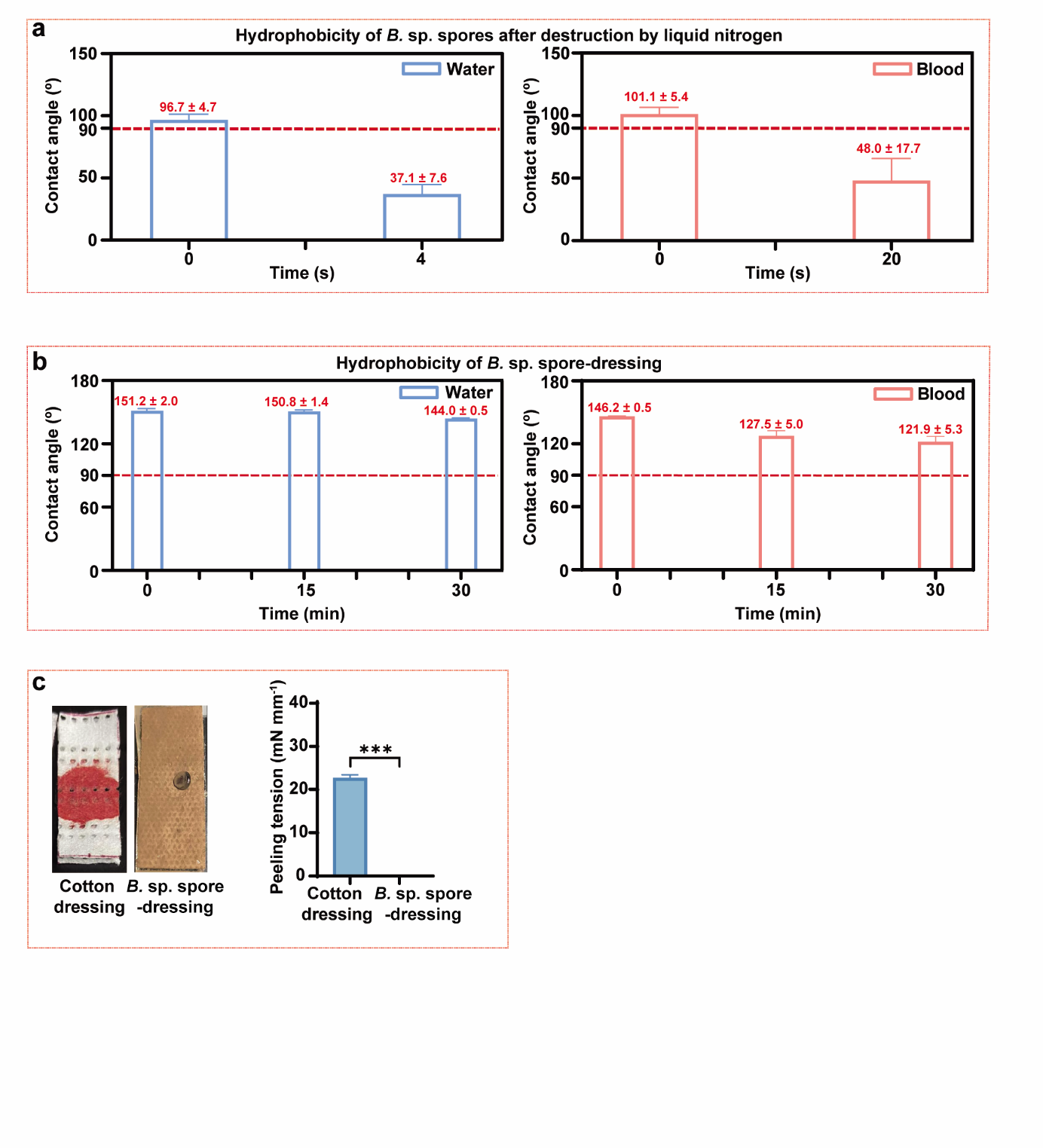


**Figure S4. Hydrophobic property of *B.* sp. spores after destruction by liquid nitrogen and *B.* sp. spore-dressing.**

(a) Water and blood contact angle of *B.* sp. spores after destruction by liquid nitrogen. (b) Water and blood contact angle of *B.* sp. spore-dressing. Data are presented as mean ± SD (*n* = 3). (c) Photograph and histograms of the clot peeling tension of *B.* sp. spore-dressing. Data are presented as mean ± SD (*n* = 3). ****p* < 0.001 by unpaired student’s *t*-test.


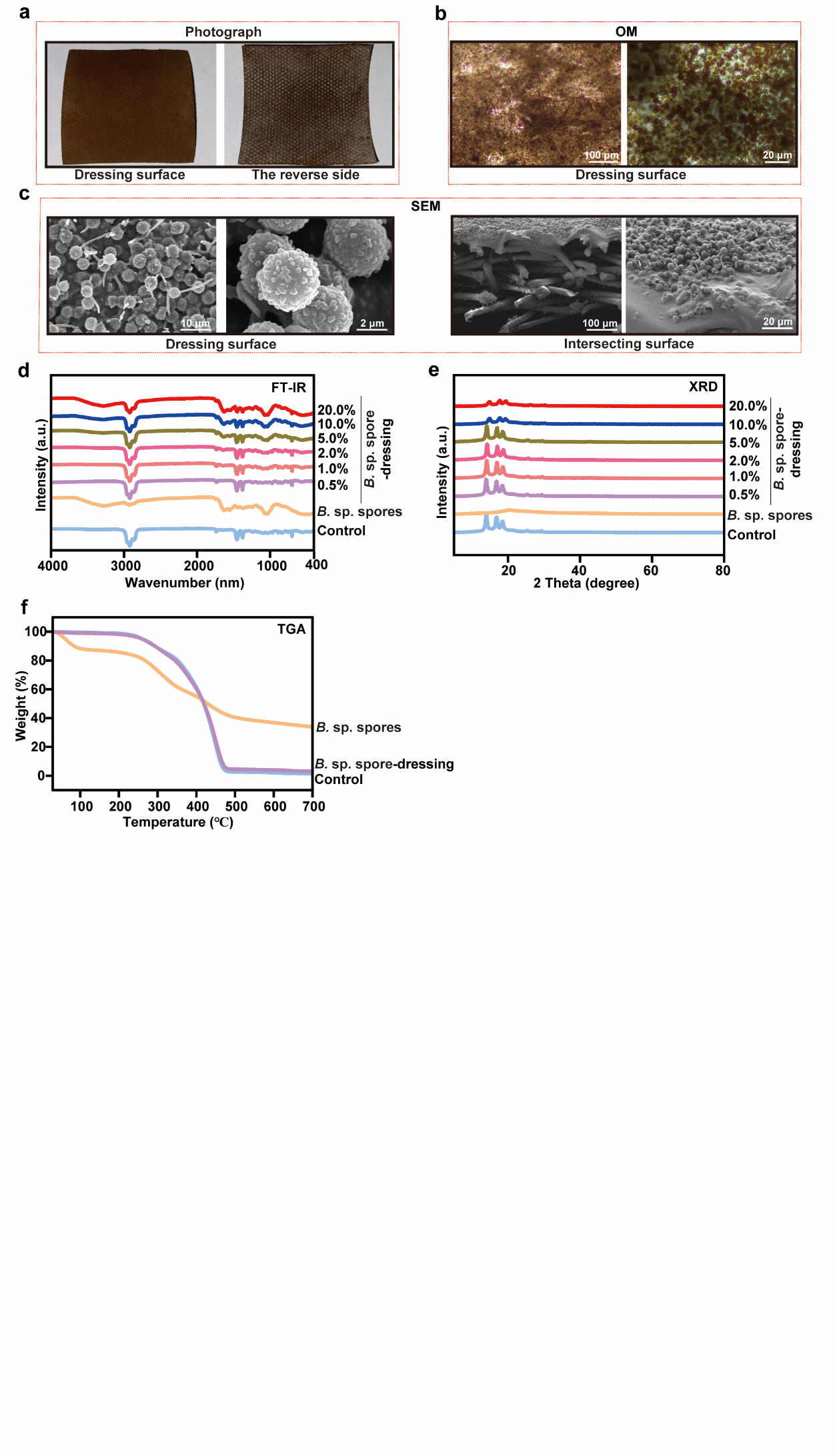


**Figure S5. Characterization of *B.* sp. spore-dressing.**

(a) Photographs of *B.* sp. spore-dressing. (b) OM images of *B.* sp. spore-dressing. (c) FE-SEM images of *B.* sp. spore-dressing. (d) FT-IR spectra. (e) XRD patterns. (f) TGA curves as well as the derivative weight as a function of temperature.

**Supplemental tables**

**Table S1.** Brunauer–Emmett–Teller (BET) measurement of *B.* sp. spores.

| BET Surface Area  [m^2^ g^-1^] | Micropore Volume  [cc g^-1^] | Micropore Area  [m^2^ g^-1^] | External Surface Area  [m^2^ g^-1^] | Pore Diameter Dv(d)  [nm] |
| --- | --- | --- | --- | --- |
| 6.67 | 0.001 | 2.21 | 4.46 | 1.45 |

**Table S2**. The weights of key metal elements (Ca, Mg, Fe) in three samples: water (control), water extract from *B.* sp. spores, and *B.* sp. spores, as well as the release rate of these elements from *B.* sp. spores to water.

| Key metal element | Key metal element weight in 25 mL water (m_A_, μg) | Key metal element weight in  25 mL water extract from  50 mg *B.* sp. spores (m_B_, μg) | Key metal element weight in 50 mg *B.* sp. spores  (m_C_, μg) | Release rate of key metal element from *B.* sp. spores  in water |
| --- | --- | --- | --- | --- |
| Ca | 24.1 | 29.4 | 66.9 | 7.9% |
| Mg | 3.1 | 31.7 | 64.5 | 44.3% |
| Fe | 1.8 | 4.0 | 19.0 | 11.7% |

(*n* = 3), data expressed as mean

**Table S3.** Thromboelastography (TEG) parameters.

| Samples | | R  [min] | K  [min] | α  [deg] | MA  [mm] | LY30  [%] |
| --- | --- | --- | --- | --- | --- | --- |
| Control | | 8.73 ± 0.32 | 2.20 ± 0.00 | 58.33 ± 0.70 | 58.43 ± 0.75 | 0.87 ± 0.20 |
| *B.* sp. spores  [mg mL^-1^] | 1 | 4.83 ± 0.35 | 1.57 ± 0.21 | 65.70 ± 2.07 | 62.43 ± 0.40 | 0.90 ± 0.35 |
|  | 2 | 4.47 ± 0.64 | 1.37 ± 0.15 | 68.00 ± 0.96 | 61.60 ± 1.45 | 0.63 ± 0.37 |
|  | 4 | 3.27 ± 0.55 | 1.37 ± 0.21 | 68.47 ± 1.66 | 60.70 ± 1.51 | 0.33 ± 0.57 |

^a^ (*n* = 3); Data expressed as mean ± SD.

**Supplementary results**

1. **Characterization results of *B.* sp. spores**

*The Fourier Transform infrared spectroscopy (FTIR) of* *B.* sp. spores: Herein, the presence of peaks at 3376 cm^-1^ in the spectrum of *B.* sp. spores (Figure S3g) may correspond to either -OH stretching vibrations or N-H stretching vibrations. The peaks at 1446 cm^-1^ and 1397 cm^-1^ correspond to the bending vibration absorption of CH_2_/CH_3_ groups. Furthermore, the peaks at 1075 cm^-1^ and 1031 cm^-1^ indicate the absorption of stretching vibrations of C-O-C, which represent the specific absorption peaks of polysaccharides. Additionally, the peak at 1069 cm^-1^ indicates asymmetric stretching of C-O-C in saccharide ring. In addition, the presence of peaks at 1742 cm^-1^, 2921 cm^-1^, and 2857 cm^-1^ corresponds to C=O and C-H stretching vibrations, consistent with lipid-specific absorption. Moreover, the absorption peaks at 1553 cm^-1^ and 1634 cm^-1^, align with Amide II band and Amide I band absorption peaks of proteins. In addition, the presence of a peak at 1147 cm^-1^ indicates the presence of asymmetric bridge oxygen stretching, aligning with chitin absorption peaks.

*The Brunauer–Emmett–Teller (BET)* *surface area measurement of* *B.* sp. spores: It indicated a BET surface area value of 6.67 m^2^ g^-1^ for *B.* sp. spores, with an exteranal surface area of 4.46 m^2^ g^-1^ and a micropore area of 2.21 m^2^ g^-1^ in Table S1. The micropore area, constituting nearly one-third of the BET surface area value, suggests the hollow nature of *B.* sp. spores.

*The wetting properties of the crushed residue (B. sp. spores after destruction by liquid nitrogen) towards water and blood*: The results were evaluated using dynamic contact angle (Figure S4a). Initially, the contact angle of water on the crushed residue was 96.7 ± 4.7°, which rapidly decreased to 37.1 ± 7.6°. Similarly, the contact angle of blood on the crushed residue was initially 101.1 ± 5.4°, which rapidly decreased to 48.0 ± 17.7°. These findings suggest that the hydrophobic property of *B.* sp. spores can be attributed to nano-protrusions on their surface.

*The sporewall structure of B.* sp. spores: To further elucidate the sporewall structure of *B.* sp. spores, ultrathin sections were produced to took TEM images (Figure 3g and Figure S3c). Figure S3c depicts the whole cross-section of *B.* sp. spores spore part and Figure 3g depicts that the spore wall consists of three layers. The color of layers 1# and 3# is darker, and the color of layer 2# is lighter under TEM. The sporophore part also has three layers. Then these ultrathin sections were stained with fluorescent brightener 28, co-stained with fluorescent brightener 28 (405 nm excitation, 422 nm emission) and auramine O (498 nm excitation, 517 nm emission), respectively. Figure S3d depicts blue double concentric ring structure, indicating that the layers 1# and 3# of *B.* sp. spores are chitin. Figure S3e also depicts a bright green ring structure consistent with the position of layer 2#, indicating that the layer 2# is composed of sporopollenin. Figure S3f depicts the position of chitin (blue, layers 1# and 3#) and sporopollenin (green, layer 2#) on *B.* sp. spores, simultaneously. In addition, there is an enrichment site of chitin near the sporophore part, and another at the top of the spore part near the symmetry. Energy dispersive spectroscopy (EDS) mapping analysis images in Figures S2h-i depict enrichment of calcium, magnesium, and iron in layers 1# and 3#, with a deficiency in layer 2#.

1. **Characterization results of *B.* sp. spore-dressing**

Photographs of the *B.* sp. spore-dressing are shown in **Figure S5**a. The optical microscopy (OM) images (Figure S5b) and SEM images (Figure S5c) revealed that the *B.* sp. spores were evenly attached to the dressing base. Additionally, FT-IR, X-ray Diffraction (XRD), and Thermogravimetric Analysis (TGA) (Figure S5d-f) demonstrated the stable binding of various amounts of *B.* sp. spores to the dressing base.

The contact angle of water on *B.* sp. spore-dressing was measured to be 151.2 ± 2.0° initially, which decreased to 144.0 ± 0.5° after 30 min (Figure S4b). On the other hand, the contact angle of blood on the *B.* sp. spore-dressing was measured to be 146.2 ± 0.5° initially, which decreased to 121.9 ± 5.3° within 30 min. The hydrophobic nature of dressing surface proves advantageous for effective hemostatic stripping at the wound site. Accordingly, clot peeling tension was measured to assess the "ease to peel" performance of *B.* sp. spore-dressing.

*Measurement of the clot peeling tension*: Two pieces of dressing were soaked in sheep whole blood and left overnight. Subsequently, the dressings were carefully removed from one side. As shown in Figure S4c, the *B.* sp. spore-dressing was easier to peel than the cotton dressing. As shown in Figure 3c, using 3M artificial skin (3M Tegaderm hydrocolloid thin dressing) as a base, excessive sheep whole blood was added between the artificial skin and the *B.* sp. spore-dressing or three commercial easy-to-peel dressings [Smith & Nephew Opsite dressing (1$), Mannings adhesive waterproof dressing (2$), and 3M Nexcare DUO dressing (3$)]. The mixture was left overnight. Subsequently, the dressings were carefully removed from one side. The adhesion evaluation involved testing the peak peel forces using a tension meter with a range of 1000 mN and an accuracy of 0.01 mN.

1. **In vivo hemostatic results of *B.* sp. spore-dressing**

The present study examined the hemostatic properties of *B.* sp. spore-dressing on epidermal trauma using a rat back skin injury model (Figure 2j-l, Figure S2). The schematic representation of the rat back skin injury model is depicted in Figure 2j, with additional photographs illustrating the general gauze (control group) and *B.* sp. spore-dressing during the initial bleeding phase shown in Figure 2k*.* The blood stain images clearly demonstrated that the application of *B.* sp. spore-dressing led to a substantial reduction in blood loss compared to the control group (Figure 2l). Moreover, the blood loss for the control group and the *B.* sp. spore-dressing was measured at 24.23 ± 4.20 mg and 17.20 ± 4.28 mg (Figure S2b), respectively. After a 3-min duration of bleeding, the dressings were removed by gentle peeling. As observed, the control group experienced continuous bleeding, while the *B.* sp. spore-dressing group exhibited accelerated clotting, resulted in the formation of a scab on the wound and effectively blocking any further blood flow (Figure S2a). The dressings were then left in place for approximately 2 h before removed. The wound site of the control group exhibited the presence of exudate, while no apparent exudate was observed at the wound site of the *B.* sp. spore-dressing group (Figure S2c). Consequently, the wounds were delicately cleansed with paper, and the state of the exudate was depicted in Figure S2d. Overall, these findings suggest that *B.* sp. spore-dressing effectively obstructs blood flow, expedites the process of blood clotting, minimizes blood loss, and exhibits minimal secondary damage in the latter scenario.

1. **Hemostatic mechanism results of *B.* sp. spores**

The *B.* sp. spores investigated in this study exhibit a negatively charged. Interestingly, it has been reported that the presence of a negative surface charge can stimulate the activation of coagulation factor XII, initiating a series of reactions in the body's natural clotting process to achieve its hemostatic effect.^[1]^ In addition, some metal ions act as important procoagulant factors in the blood coagulation process, playing a key role in regulating the activity of thrombin and other coagulation factors. For instance, calcium ions (Ca^2+^) has been demonstrated to play a crucial role in the coagulation process as coagulation factor IV.^[2]^ Furthermore, magnesium ions (Mg^2+^) can enhance the binding of Ca^2+^ to coagulation factor IX, thereby initiating the coagulation cascade.^[3]^ Additionally, Mg^2+^ can reinforce the interaction between tissue factor and X factor, further promoting clotting.^[4]^ Iron ions (Fe^3+^) promote translocation of procoagulant platelet to accelerates thrombosis in hemostasis.^[5]^ Thus, we investigated the concentrations of these key metal elements (calcium, magnesium, and iron) in *B*. sp. spores and their respective release rates in water. The results revealed that the contents of calcium, magnesium, and iron in the spores were approximately 1.34 mg/g, 1.29 mg/g, and 0.38 mg/g, respectively. Furthermore, their release rates in water were measured to be about 7.9%, 44.3%, and 11.7%, respectively. These findings demonstrate that *B.* sp. spores can facilitate hemostasis not only through their negatively charged surface but also via the release of calcium, magnesium, and iron ions.

1. **Biocompatibility results of *B.* sp. spores and *B.* sp. spore-dressing**

The blood compatibility test indicated that both *B.* sp. spores and *B.* sp. spore-dressing exhibited low levels of hemolysis (Figure 5a and 5c). In addition, CCK-8 assay in mouse fibroblast cell line L929 suggests that *B.* sp. spores leachate did not induce cytotoxicity (Figure 5b). *B.* sp. spore-dressing leachate at various concentrations also had minimal impact on cell viability (Figure 5d). The AO/EB staining method, which highlights dead cells wtih EB fluorescence in an orange-red color, was also utilized to confirm the safety of *B.* sp. spore-dressing. These images confirmed that *B.* sp. spore-dressing did not exhibit cytotoxicity (Figure 5e). Furthermore, the SD rat back skin irritation experiment was utilized to evaluate the safety in vivo. No obvious skin irritations (pruritus, erythema, or swelling) were observed within a 24-h period. Subsequently, the dermal tissue was collected for histological examination using hematoxylin and eosin (H&E) staining. Accordingly, no inflammation was observed in both the dressing group and the control group (Figure 5f). Thus, the experimental results demonstrated that both the *B.* sp. spores and the *B.* sp. spore-dressing exhibit excellent biocompatibility.

**Supplementary materials and methods**

1. **General experimental procedures**

Basewing dressing was purchased from Basewing Medical Co., Ltd., China. Cotton dressing was purchased from Winner Medical Co., Ltd., China. The Smith & Nephew Opsite dressing was obtained from Smith & Nephew, Inc., UK. Mannings adhesive waterproof dressing was obtained from MANNINGS, Ltd., China. 3M Nexcare DUO dressing was obtained from 3M China, Ltd., China. The QuikClot™ Combat Gauze was acquired from Chinook Medical Gear, Inc., USA. Additionally, the QuikClot™ powder was produced by saturating QuikClot™ Combat Gauze with distilled water, then filtering the resulting solution and subsequently drying it.

Sheep whole blood, anticoagulated with sodium citrate at a ratio of 1:9 was obtained from Guangzhou Hongquan Biological Technology Co., Ltd., China. Fluorescent brightener 28 was purchased from Shanghai Macklin Biochemical Technology Co., Ltd., China. Auramine O was purchased from Shanghai Aladdin Biochemical Technology Co.,Ltd., China. Zoletil 50 was purchased from The Virbac Group, France. Su-Mian-Xin was purchased from Dunhua City Shengda Animal Medicine Co., Ltd, China. The L929 cell line was acquired from the American Type Culture Collection (USA). Cell culture reagents were procured from Thermo Fisher Scientific (USA). The staining solution, consisting of Acridine orange and Ethidium bromide (AO/EB), was acquired from Beijing Solarbio Science & Technology Co., Ltd., China. The Cell Counting Kit-8 (CCK-8) reagents were obtained from Dojindo Laboratories, Japan. Ultrapure water was prepared using a Milli-Q Integral Water Purification System, Germany. The Activated partial thromboplastin time (APTT) kit was procured from Beijing Leagene Biotechnology Co., Ltd., China. The simulated blood was procured from Beijing Solarbio Science & Technology Co., Ltd., China. Syringe Filters (0.22 µm/28 mm, PES, Sterile) were acquired from Sartorius Corporate Administration GmbH, Germany. The electric vibrating screen machine (hy-zsj-8411) was acquired from Guangzhou Huayu Trading Co., Ltd., China.

Prior to evaluating their biological performance, all materials were subjects to sterilization via ultraviolet radiation or moist-heat sterilization.

1. **Fungus material and ethic information**

The puffball specimen (PBHLJ210901) was obtained from Shuang Yashan City, located in Heilongjiang Province, China. The fungus was identified as *Bovistella* sp. based on its morphological characteristics and ribosomal internal transcribed spacer (ITS):

ACTATTGAAATTCTTGATGGGTTGTAGCTGGCTCTTCGGGGCATGTGCACACTTGTCTTGACTTTATTCATCCACCTGTGCACCTTTTGTAGTCTTGGGGGTTGAGAGCAGTCGACTATCGGATGGCTATGGCCTTTCCGGACGTGAGGAATGCTGAGTGCGAAAGCATACAGCTCTTCTCAAATGACTTGCAAAACCTCTCCCTCGAGTACTATGTATTCATATACCACATCGTATGTTGTAGAATGTGATCAATGGGCCTATGTGCCTATAAAAATCATATACAACTTTCAGCAACGGATCTCTTGGCTCTCGCATCGATGAAGAACGCAGCGAAATGCGATAAGTAATGTGAATTGCAGAATTCAGTGAATCATCGAATCTTTGAACGCACCTTGCGCTCCTTGGTATTCCGAGGAGCATGCCTGTTTGAGTGTCATTAAATTCTCAACCCCTCCAGCTTTTGCGAGTTGTGATGGGGCTTGGATATGGGAGTTTGCGGGTCTTTATCAATAAAGGTCAGCTCTCCTGAAATACATTAGCGGAACCGTTTGCAGTCCCGTCACTAGTGTGATAATTATCTACACTGTGATGATTGCTCTCTGACTAGTTCAGCTGCTAATCGTCCACTATGGACAACACTTAATGAACTTCTTGACCTCAAATCAGGTAGGACTACCCGCTGAACTTAAGCATATCATAAAAGCGGAGGGAAAGGGGGGGGTCTCTCTAG

SD rats were purchased from SPF (Beijing) Biotechnology Co., Ltd., SPF grade, aged 6-8 weeks and weighing 180~200 g. The experiment protocol was endorsed by the Animal Welfare Ethics Committee of Jinan University, with IACUC approval No.20220704-08. After introduction, the animals were fed at a density of 5 animals per cage. New Zealand rabbits were purchased from Longgui Xingke animal Farm, Baiyun District, Guangzhou by Guangdong Huawei Detection Co., LTD., ordinary grade, weighing 1.8~2.2 kg, with an equal ratio of males and females. The experiment was approved by the experimental Animal Ethics Committee of Guangdong Huawei Testing Company. The institutional animal care and use committee (IACUC) issue is No.202209005. Following their introduction, the animals were single housed and fed. All animal experiments adhered to the 3R principle, respecting the life and rights of animals, and giving corresponding animal welfare. The blood used to study the mechanism of hemostasis in the experiment was freshly collected from the elbow veins of healthy volunteers. The experiment was approved by the Medical Ethics Committee of Jinan University, with the approval letter numbered No. JNUKY-2023-0117.

1. **The preparation of *B.* sp. spores and *B.* sp. spore-dressing**

The puffball specimen (PBHLJ210901) was harvested at maturity and subsequently dissected. The spores were derived from the contents of *Bovistella* sp. and filtered using a 300 meshes sieve. The resultant *B.* sp. spores were then added to a Basewing® dressing to generate *B.* sp. spore-dressing using a ${\text{W}_{\text{B.}\text{ }\text{sp. spores}}}/{\text{W}_{\text{Basewing}\text{}\text{ dressing}}\text{ }}$ratio of 20%. The procedure for preparing *B.* sp. spores and *B.* sp. spore-dressing is illustrated in Figure S1a. The weight ratio of *B.* sp. spores to dressing was optimized to be 20% based on the in vitro hemostatic capacity of the *B.* sp. spore-dressing (Figure S1b).

1. **Characterization of *B.* sp. spores**

The dried *B.* sp. spores were analysed using transmission electron microscopy (TEM) instrument (JEM, 1400 Flash) operating at an acceleration voltage of 100 kV, and field emission scanning electron microscopy (FE-SEM) (ULTRA55, Carl Zeiss Jena Co. Ltd., Germany) after applying a 5 nm thick layer of gold via sputter-coating. The *B.* sp. spores were dispersed in pure water and deposited onto a mica sheet for atomic force microscopy (AFM) analysis using (BioScope Catalyst NanoScope V from Bruker Instruments Ltd., USA). The ultrathin sections was obtained using Leica EM UC7. TEM images were captured using FEI Talos F200X. Confocal images were acquired using Laser Scanning Confocal Microscope (Carl Zeiss,LSM800). EDS mapping analysis was determined using FEI Talos F200X (SuperX 4 SDD). The *B.* sp. spores were dried, mixed, and pulverized with KBr. The resulting mixture was then compressed into tablets for infrared (IR) spectroscopy (FT-IR 4600 plus, Jasco International Co. Ltd., Japan). *B.* sp. spores were dispersed in ultrapure water for analyzing zeta (ζ) potential (Nano ZS zeta potential analyzer, Malvern Instruments Co., UK). In addition, the *B.* sp. spores were dried and utilized to acquire the Brunauer–Emmett–Teller (BET) data using the Quantachrome Autosorb iQ3 [Anton Paar (Shanghai) Trading Co. Ltd., China].

The *B.* sp. spores were combined with ultrapure water until the powder was completely saturated. The excess water was removed with a straw. The water absorption amount was then calculated using the following formula:

$\text{Weight}_{\text{after}}\text{-}\text{Weight}_{\text{before}}$………….………….………….………….………….………….….….………….………….………….………….……………(1)

The ultrapure water used in the previous steps was substituted with citrated sheep whole blood in order to determine the blood absorption volume of *B.* sp. spores. The contact angle of water and citrated sheep whole blood was measured on the *B.* sp. spores tablets using a contact angle tester. Furthermore, *B.* sp. spores were pulverized and fragmented in the presence of liquid nitrogen. The water and citrated sheep whole blood onto the tablet using an optical contact angle measuring instrument (Beituo (Guangzhou) Technology Co. Ltd., China) to collect data.

1. **Characterization of *B.* sp. spore-dressing**

A type of dressing that was coated with the *B.* sp. spores (*B.* sp. spore-dressing) was created in this study. The procedure for preparing the *B.* sp. spore-dressing is illustrated in Figure S1a. *Determine the coating ratio*: A 1.5 mL centrifuge tube was used to add for blank, *B.* sp. spores (1mg) or *B.* sp. spore-dressings (5 mg) varied in weight rotio of *B.* sp. spores/dressing, ranging from 0.5%, 1%, 2%, 5%, 10%, and 20%, respectively. The tubes were filled with whole blood (100 µL) and CaCl_2_ (10 µL, 0.2 M). 1 mL of deionized water was added to the centrifuge tubes at 0, 10, and 12 min, respectively, in order to release the free blood components. The supernatant's absorbance at 540 nm was quantified using a microplate reader (Tecan/Spark 10M, Tecan Trading AG, Switzerland). Greater absorbance indicates a less adequate coagulation state, implying a diminished coagulation-promoting effect of the sample.

Optical microscopy (OM) photographs of the *B.* sp. spore-dressing were captured using a BX53 microscope (Olympus, Japan). Moreover, the surface morphology of the *B.* sp. spore-dressing was examined using a field emission scanning electron microscope (FE-SEM) (ULTRA55, Carl Zeiss Jena Co. Ltd., Germany) after applying a 5 nm thick layer of gold through sputter-coating. Additionally, the Fourier-transform infrared (FTIR) spectra were acquired through attenuated total reflectance using a spectrometer (Nicolet iS50, Thermo Fisher Scientific Co. Ltd., USA), while X-ray diffraction (XRD) patterns were obtained using a Miniflex 600 diffractometer (Rigaku Corporation, Japan) operating at a current of 40 mA and a voltage of 40 kV. Additionally, the thermal stability of the materials was also assessed through Thermogravimetric analysis (TGA) using a TGA2 instrument (Mettler Toledo, Co. Ltd., Switzerland).

1. **In vitro hemostatic assay**

*Determination of whole blood clotting time assay*: The experiment involved preheating EP tubes (2.0 mL) at 37℃. Then, citrated sheep whole blood (1 mL) was taken and added to the preheated EP tube. Varying amounts of *B.* sp. spores (0, 1, 2, 4, 8, and 16 mg) were added into separate preheated EP tubes. Finally, calcification reagent CaCl_2_ (100 μL, 0.2M), was added to each EP tube to initiate coagulation. The tubes were agitated and tilted at ten seconds intervals to monitor clot formation until the blood was fully coagulated, and the duration of clotting was documented.

*Vascular injury hemorrhage model*: A 7 mm × 7 mm aperture was created using an ophthalmic incision on the interior of a polyethylene hose with a diameter of 10 mm, in order to simulate the wound. The dressing and the *B.* sp. spore-dressing were affixed to the wound using medical adhesive tape. The entire vessel model was attached to a pre-weighed dish using 3M adhesive. Subsequently, 3 mL of citrated sheep whole blood was combined with CaCl_2_ (300 μL, 0.2M) to initiate coagulation was introduced into the hose. The dressing was then removed from the hose at a specific moment, and the changement in dish quality demonstrated the hemostatic impact of the dressing in this vascular model. The duration from the initiation of exudation to its cessation is referred to as the period of hemostasis. A control group, consisting of uncoated dressings, was also included in the study.

1. **In vivo hemostatic assay**

*Rabbit femoral arteriovenous injury model*: Rabbits were anaesthetized via intramuscular injection with a mixture of Zoletil 50 (0.3 mL per animal) and Su-Mian-Xin (0.2 mL per animal). They were then immobilized, a surgical procedure was performed to expose the groin area, straightening the hind limb, and removing hair. The skin of the animal was then incised using a scalpel, and the soft tissue of the thigh was gently separated to reveal the femoral artery and vein. The femoral artery was completely severed, resulting in extensive damage and uncontrollable hemorrhaging. After 10 seconds of bleeding, the powder or dressing was promptly applied to the injured site with manual pressure maintained until hemostasis was achieved, and the temperature was measured simultaneously. In the blank group, the uncontrolled blood loss amounted to 840 s without any intervention. If bleeding continued for 840 s, standard gauze pressure was employed as a treatment method. The control group was treated with conventional gauze pressure, and the dosage was adjusted to ensure effective hemostasis, with QuikClot powder, and *B.* sp. spores administered at doses of 0.1 g each. Upon completion of the study, fluid and clotting material obtained from the groin were collected and quantified using standard gauze. Survival rabbits were determined after a duration of 180 min, and the animals that survived were anesthetized and euthanized using air embolization. Hemostatic time and temperature data were recorded at 30 s-intervals throughout the study, blood loss were recorded after the experiment.

*Rat tail amputation model*: The weight of SD rats was measured, and they were then anesthetized by intramuscular injection (0.001 mL g^-1^). The anaesthetics were formed by mixing Zoletil 50 (1 mL), Su-Mian-Xin (1 mL), and normal saline (8 mL). Post-anaesthesia, a 4 cm section of the rat's tail was amputated at the distal end. The rat's tail was promptly treated with either powder or dressings. Hemostatic time were recorded at 30 s-intervals throughout the study and the blood loss was documented until the hemostasis was achieved. The control group was treated with conventional gauze pressure, and the dosage was adjusted to ensure effective hemostasis, with QuikClot powder, and *B.* sp. spores administered at doses of 0.03 g each.

*Tangential wound model*: The weight of SD rats was measured, and they were then anesthetized by intramuscular injection of 0.001 mL g^-1^. The anaesthetics solution was composed of Zoletil 50 (1 mL), Su-Mian-Xin (1 mL), and normal saline (8 mL). The rat was then placed in a prone position, immobilized, and the dorsal fur was removed. Subsequently, a 1 cm incision was made in the muscle on the rat's back. The *B.* sp. spore-dressing and control dressing were swiftly applied, and photographs of the samples were captured. At the 3-minute interval, the wound was inspected and the dressing was evaluated to quantify blood that was lost. The state of the wound and the occurrence of bleeding were observed 2 h later.

1. **Hemostatic mechanism assay**

*Thromboelastography (TEG)*: A sample of venous blood from healthy volunteers was collected with a sodium citrate to blood ratio of 1:9, and gently inverted ten times to ensure thorough homogenization. Preventing the formation of any blood clot or foam was strictly prohibited. Citrated whole blood (2 mL) was mixed with *B.* sp. spores, resulting in final concentrations of 0 (blank), 1, 2, and 4 mg mL^-1^. The blood was transferred into empty collection vessels (2 mL) and subsequently inserted into the Haema TX Automatic thromboelastogram analyser (Shenzhen Maiketian Biomedical Technology Co., LTD., China).

*Intrinsic coagulation cascade activation assay*: The APTT kit was employed for testing. The process involved spinning fresh whole blood from healthy volunteers at a speed of 3000 rpm for a period of 10 min in order to separate the upper layer, known as citrate poor platelet plasma (PPP). Prior to the experiment, all reagents and plasma were preheated to a temperature of 37℃. The *B.* sp. spores or positive control QuikClot (final concentration 1 mg mL^-1^) was evenly mixed with the phospholipid. Subsequently, PPP (1 mL) was introduced and incubated at a temperature of 37℃. To initiate clotting, an equivalent amount of CaCl_2_ (1 mL, 25 mm) was added. The EP tube was subjected to periodic vibration and then tilting at ten seconds interval, while closely observing the process of clotting until the formation of a white insoluble substance. Subsequently, the coagulation time was recorded. A decreased APTT suggests enhanced activation of the intrinsic clotting cascade.

*Analyses of key metal element (Ca, Mg, Fe) weights in water (control), water extract from B. sp. spores, and B. sp. spores*: The experiment was divided into three groups: group A (25 mL water), group B (water extract from *B.* sp. spores, 25 mL water + 50 mg *B.* sp. spores), and group C (50 mg *B.* sp. spores). The samples of groups A and B were incubated at 37 ℃ and 200 rpm for 30 min. Subsequently, the samples were filtered using membrane filters with a pore size of 0.22 µm. The samples were dried, weighed, and their weights were recorded. The obtained filtrate samples from groups A and B, along with 50 mg *B.* sp. spores from group C were digested using nitric acid with the aid of ultrasonic wave. Then, the key metal element (Ca, Mg, Fe) weights of these samples were measured using an inductively coupled plasma optical emission spectrometer (ICP-OES; Perkin Elmer Instruments Co., Ltd, USA). The release rate of key metal element from *B*. sp. spores into water was calculated using the following formula:

Release rate of key metal element from *B.* sp. spores to water (%) = (m_B_-m_A_)/m_C_ × 100%.………….………….……………….………(2)

1. **Biocompatibility assay**

*Blood compatibility evaluation*: Phosphate-buffered saline (PBS, 10 mL) was mixed with citrate-treated sheep whole blood (1 mL). It was then centrifuged thrice at 500 g for 10 min to isolate erythrocytes from the blood. The isolated red blood cells (200 μL) were then added to a sample suspension (800 μL) obtained from dressing volumes of 37.5, 75, 150, 300, 600, and 1200 μg mL^-1^. The sample suspension was created by combining the *B.* sp. spores or *B.* sp. spore-dressing fragments with PBS. The positive control was composed of ultra-pure water with red blood cells, while the blank control consisted of PBS with red blood cells. The samples were subjected to incubation at a temperature of 37℃ for a duration of 2 h and centrifuged at 1000 g for a period of 5 min to separate and collect the red blood cells. Subsequently, the absorbance was assessed at a wavelength of 540 nm. The rate of hemolysis was then determined using the following formula:

$\text{Hemolysis rate }\left( \text{\%} \right)\text{ = }\frac{\text{OD}_{\text{sample}}\text{-}\text{OD}_{\text{blank}}}{\text{OD}_{\text{positive}}\text{-}\text{OD}_{\text{blank}}}\text{×100\%}$.………….………….………….…….………….………….………….………………..………….………(3)

*Evaluation of cytotoxicity*: The evaluation of cell death was conducted by examining the cytotoxic effects on the L929 mouse fibroblast cell line. The cells were cultivated in high glucose DMEM, supplemented with FBS (10%) and penicillin-streptomycin solution (1%), in an environment containing 5% CO_2_ at a temperature of 37℃. The presence of viable cells can be determined through morphological evidence using AO/EB staining. The autoclaved *B.* sp. spores or crushed *B.* sp. spore-dressing was immersed in complete medium and left overnight to obtain 100% leachate (10 mg mL^-1^). The *B.* sp. spores or *B.* sp. spore-dressing was then prepared by submerging them in a medium with a gradient ratio of 0% and 20%, 40%, 60%, 80%, or 100% material leachate. L929 cells (3 × 10^4^ cells mL^-1^) in the logarithmic growth phase were added to 96-well plates (100 μL well^-1^). The cells were then treated with material leachate after 6–8 h. After a 48-hour interval, the cellular morphology was observed using a live cell imaging workstation (Observer Z1, Carl Zeiss Microscopy GmbH, Germany) following staining with AO/EB*.* Additionally, cell viability was assessed using the CCK-8 assay and the absorbance was measured at 450 nm using a microplate reader (Tecan/Spark 10M, Tecan Trading AG, Switzerland).

*Skin irritation test*: The weight of SD rats was measured, and they were then anesthetized by intramuscular injection (0.001 mL g^-1^). The anesthetic solution was prepared by mixing Zoletil 50 (1 mL), Su-Mian-Xin (1 mL), and normal saline (8 mL). The rat was then placed in a prone position, immobilized, and the dorsal fur was removed. The dorsal skin was then covered with the *B.* sp. spore-dressing or control dressing for 24 h, and erythema, pruritus, and cracking were observed. Subsequently, a section of the skin (10 × 10 mm) was extracted from the test area and preserved in a 4% paraformaldehyde solution for a duration of 2 days. The paraffin-embedded sections underwent hematoxylin-eosin (H&E) staining at Wuhan Servicebio Technology Co., Ltd., China.

**Statistical analysis**

All data were shown as means ± SD or mean via at least triplicate samples. The detailed n values for each panel in the figures are provided in the corresponding legends. GraphPad Prism version 8 was employed to draw graphs. The data were analyzed by IBM SPSS Statistics 26.0 (SPSS Inc., Chicago, IL, USA). Two-tailed unpaired student’s *t*-tests were utilized for comparison involving the two groups. More than two groups were compared separately by one-way ANOVA (analysis of variance) using a Tukey post-hoc analysis. **p* < 0.05, ***p* < 0.01, ****p* < 0.001 denote statistically significant variation.

**References**

[1] M. Fischer, C. Sperling, C. Werner, *Journal of Materials Science: Materials in Medicine*, **2010**, *21*, 931-937.

[2] W. Xiong, Y. Zhao, Y. Xiong, M. Xu, B. Pudasaini, H. Du, X. Guo, *The Clinical Respiratory Journal*, **2020**, *14*, 124–131.

[3] F. Sekiya, T. Yamashita, H. Atoda, Y. Komiyama, T. Morita, *The Journal of biological chemistry*, **1995**, *270*, 14325-14331.

[4] J. M. Gajsiewicz, K. M. Nuzzio, C. M. Rienstra, J. H. Morrissey, *Biochemistry*, **2015**, *54*, 4665-4671.

[5] a) D. Y. Nechipurenko, N. Receveur, A. O. Yakimenko, T. O. Shepelyuk, A. A. Yakusheva, R. R. Kerimov, S. I. Obydennyy, A. Eckly, C. Léon, C. Gachet, E. L. Grishchuk, F. I. Ataullakhanov, P. H. Mangin, M. A. Panteleev, *Arteriosclerosis, Thrombosis, and Vascular Biology*, **2019**, *39*, 37-47; b) C. Lv, L. Li, Z. Jiao, H. Yan, Z. Wang, Z. Wu, M. Guo, Y. Wang, P. Zhang, *Bioactive Materials*, **2021**, *6*, 2346-2359.

# Supplementary Videos

**1**. Rapid hemostatic of a bleeding in rabbit femoral arteriovenous injury model by Blank.

**2**. Rapid hemostatic of a bleeding in rabbit femoral arteriovenous injury model by Control (conventional gauze).

**3**. Rapid hemostatic of a bleeding in rabbit femoral arteriovenous injury model by QuikClot™ powder.

**4**. Rapid hemostatic of a bleeding in rabbit femoral arteriovenous injury model by *B.* sp. spores.

**5**. Rapid hemostatic of a bleeding in rabbit femoral arteriovenous injury model by QuikClot™ COMBAT GAUZE.

**6**. Rapid hemostatic of a bleeding in rabbit femoral arteriovenous injury model by *B.* sp. spore-dressing.

**7**. Observation of water absorption by *B.* sp. spores under a biomicroscope.

**8**. Observation of water absorption by *B.* sp. spores from blood under a biomicroscope.

**9.** Observation of water absorption by *B.* sp. spores.

**10.** Observation of water absorption by *B.* sp. spore-dressing.
